# Morphological, Biochemical and Molecular Characterization of Twelve Nitrogen-Fixing Bacteria and Their Response to Various Zinc Concentration

**DOI:** 10.5812/jjm.9415

**Published:** 2014-04-01

**Authors:** Mohammad Dadook, Sedigheh Mehrabian, Mitra Salehi, Saeed Irian

**Affiliations:** 1 Faculty of Biological Science, Islamic Azad University Tehran North Branch, Tehran, IR Iran; 2 Department of Cell and Molecular Biology, Faculty of Biological Sciences, Kharazmi University, Tehran, IR Iran

**Keywords:** Zinc, *Azotobacter*, nifH Gene

## Abstract

**Background:**

Zinc is an essential micronutrient used in the form of zinc sulfate in fertilizers in the agriculture production system. Nitrogen-fixing microorganisms are also of considerable value in promoting soil fertility.

**Objectives:**

This study aimed to investigate the degree of sensitivity to varying concentrations of zinc, in the form of ZnSO4, in different strains of *Azotobacter chroococcum* in a laboratory environment.

**Materials and Methods:**

To isolate *A. chroococcum* strains, soil samples were collected from wheat, corn and asparagus rhizospheres and cultured in media lacking nitrogen at 30˚C for 48 hours. Strains were identified based on morphological and biochemical characteristics. The presence of the nitrogenase enzyme system was confirmed by testing for the presence of the *nifH* gene using PCR analysis. The minimum inhibitory concentration (MIC) and optimal zinc concentration for the growth of each strain was determined.

**Results:**

A total of 12 bacterial strains were isolated from six different soil samples. *A. chroococcum* strains were morphologically and biochemically characterized. The presence of the *nifH* gene was confirmed in all the strains. MIC and the optimal zinc concentration for bacterial growth were 50 ppm and 20 ppm, respectively.

**Conclusions:**

It was concluded that increasing the concentration of zinc in the agricultural soil is harmful to beneficial microorganisms and reduces the soil fertility. A 20-ppm zinc concentration in soil is suggested to be optimal.

## 1. Background

Heavy metals are elements with an atomic mass greater than 40 g and a specific weight of more than 5 g/cm^3^ (1). These elements often find their way into soil through environmental contaminants including the atmospheric pollutants in the industrial regions, unlimited use of agricultural fertilizers, and municipal and industrial sewage system in a nonreturnable fashion (2). Unlike organic contaminants which can be converted to nontoxic compounds, metals are intrinsically stable in nature (3). Certain metals including zinc (Zn) are essential for plant growth and development, when used as a micronutrient, however, when used in greater amounts, may result in metabolic disorders, eventually suppress the growth of most plants and microorganisms (4). Generating free radicals and oxidative stress are important mechanisms that heavy metals, including zinc apply to induce toxicity (5).

In general, variation and distribution of microorganisms is a reflection of soil fertility. As nitrogen is an essential element for plants, nitrogen-fixing bacteria would provide molecular nitrogen for plant use (6). *Azotobacter* species belong to the Gram-negative and the polymorphic family of *Azotobacteraceae* are capable to forming capsule and microcyst (7). By fixing nitrogen and producing thiamin, riboflavin, nicotin, indole-3-acetic acid (IAA) and gibberellin, these bacteria participate in plant cell growth (8). Molecular nitrogen is converted to ammonia by the nitrogenase in the biological nitrogen fixation process. Synthesis of a functional nitrogenase requires the expression of *nif* genes. The structural gene *nifH*, as an important *nif* gene, is involved in the formation of Fe-protein complex (9). Nitrogen-fixing bacteria can now be detected based on the presence of *nif* gene using PCR or sequencing techniques (10).

## 2. Objectives 

Due to the key role played by the nitrogen-fixing microorganisms in the agricultural soil, and the importance of the metal zinc as an essential micronutrient in biological cycles along with its toxic effect on the environment, this study aimed at investigating the degree of sensitivity to different concentrations of zinc, in the form of ZnSO_4_, in different strains of *Azotobacter chroococcum* in a laboratory environment.

## 3. Materials and Methods

All chemicals and reagents were purchased from Merck (Germany).

### 3.1. Soil Sampling

Soil samples were collected during April 2012 in Aznova Behnamir, Mazandaran province, Iran. Samples were withdrawn from 0 - 30 cm- depth surfaces, collected into sterile vials and transferred to laboratory. These included 1, 2 and 3 samples collected from wheat, corn and asparagus rhizospheres, respectively.

### 3.2. Isolation of Nitrogen-Fixing Microorganisms From Agricultural Soil

Isolation of *A. chroococcum* strains was performed in duplicates by the dilution-pour plates method (10^-1^-10^-10^) on mannitol N-free agar medium containing: mannitol 10 g, K_2_HPO_4_ 0.75 g, MgSO_4_ 0.5 g, CaCO_3_ 3 g, sodium molybdate 0.02 g, agar 18 g and H_2_O dist. 1000 mL. Samples were incubated for 48 hours at 30˚C, before being subjected to both microscopic and macroscopic analysis. Bacterial cultures were repeated three times for a better isolation and purification purpose. Identification of the strains was performed using the routine microbiological method including biochemical tests (11, 12).

### 3.3. Culture Inoculation

Isolated colonies were grown on Brain Heart Infusion (BHI) agar plates at 30˚C for 24 hours. Bacterial colonies were then inoculated into sterile physiological serum and the optical density (OD) was adjusted to 0.8-1 at 600 nm, equivalent to 5 × 10^-8^ CFU/mL.

### 3.4. Sensitivity Assay to Different Concentrations of Zinc Sulfate for Isolated Strains

To assay the sensitivity of the isolated strains to zinc sulfate, samples of 0.1-10 mM serial concentrations of ZnSO_4_^•^7H_2_O [287.34 MW] were prepared in Luria Broth (LB) medium and sterilized. Then 1 mL of the bacterial sample was added to each medium and incubated at 30˚C for 24 hours on a shaking incubator prior to measuring the OD at 600 nm. In a different approach, the sensitivity level was determined in 100 mL of LB containing 10 to 100 ppm of zinc sulfate and 1% (v/v) bacterial inoculum in 300 mL flasks. These cultures were then incubated at 30˚C for 24, 48 and 72 hours on a shaking incubator, set at 125 rpm, prior to OD reading at 600 nm. Positive (zinc-free medium containing bacteria) and negative (minimum zinc concentration with no bacteria) controls were also included.

### 

### 3.5. The Presence of *nif*H Gene

#### 3.5.1. Bacterial DNA Extraction

Isolated bacteria were cultured in LB medium on a shaking incubator at 30˚C for 18 hours. These cultures were then centrifuged at 12000 rpm for 2 minutes. Genomic DNA was extracted using the MBST DNA Extraction kit (Germany/Iran).

#### 3.5.2. Polymerase Chain Reaction

A 25-μL PCR reaction mix was prepared using primers: 5′TTCCATCAGCAGCTCTTCGA3′ and 5′GGCAAAGGTGGTATCGGTAA3′ (Robin Teb Gostar-Iran). The PCR thermo cycling condition was 95˚C for 3 minutes (preheating), 95˚C for 30 seconds, 57˚C for 30 seconds, and 72˚C for 45 seconds and 30 cycles, followed by a final heating at 72˚C for 7 minutes. The PCR product size was confirmed by electrophoresis on a 1% agarose gel (Bio-Rad-USA). All reagents, Taq polymerase and DNA ladder were purchased from Metabion (Germany).

## 4. Results

**Figure 1.**
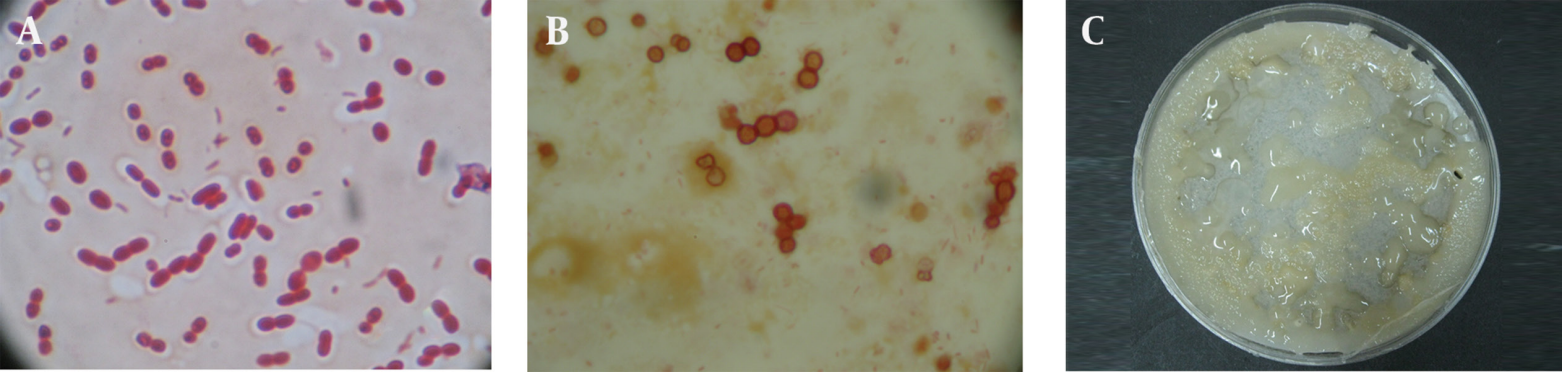
Microscopic (Light Microscope 100x) and Macroscopic Examinations of the Gram-Negative Bacilli, Capable of Forming Cyst A. Gram-negative Bacilli; B. Cyst on Mannitol N-free Agar Medium; C. Colonies o f *A. chroococcum* on Mannitol N-free Agar Medium

**Figure 2.**
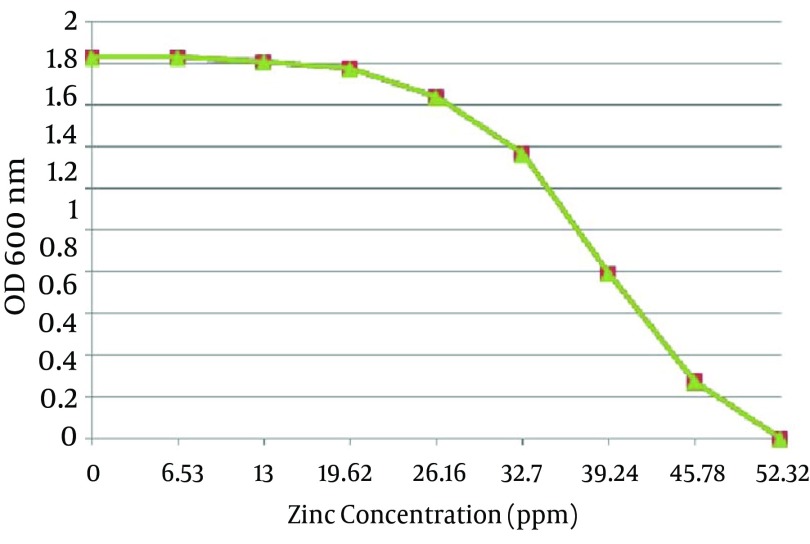
The Mean Optimal Growth Rate of Twelve *A. chroococcum* Strains at Different Concentrations of Zinc

A total of 12 *A. chroococcum* strains were identified in the agricultural soil samples collected from the rhizosphere. Microscopic and macroscopic examinations of the Gram-negative bacilli, capable to form cyst, white, transparent, viscous and moist colonies which turn dark brown after 5-7 days of incubation on a mannitol N-free agar medium, along with the biochemical tests revealed the identity of different *A. chroococcum* strains (Figure 1 and Table 1). The first method determined the MIC of all the strains as 0.8 mmol zinc sulfate (52.33 ppm), while the optimal growth rates of bacteria determined by OD analysis were 0.1, 0.2 and 0.3 mmol, equivalent to 6.53, 13 and 19.62 ppm, respectively (Figure 2). 

**Figure 3.**
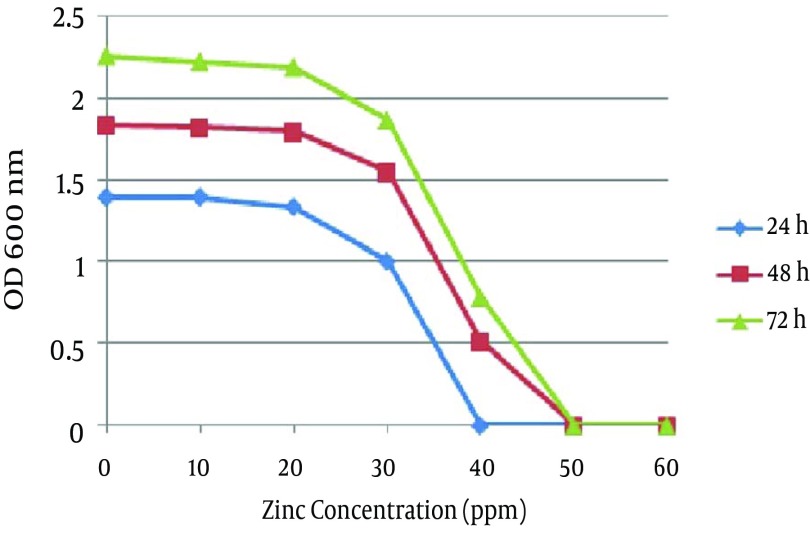
The Mean Optimal Growth Rate of Twelve *A. chroococcum* Strains at Different Concentrations of Zinc After 24, 48 and 72 Hours

**Figure 4.**
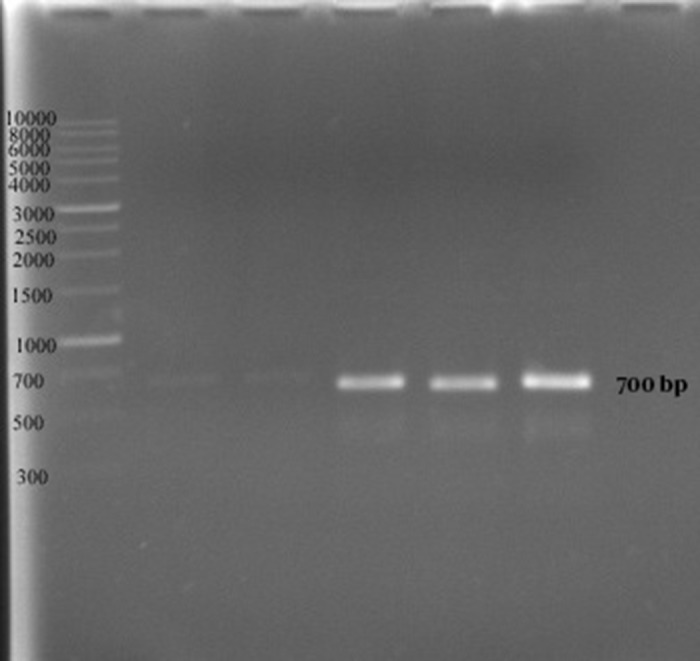
PCR Products on An Agarose Gel Image Showing the Presence of *nifH* Gene in DNA Extracted From Five A. chroococcum Strains

In the second method no growth was detected at 40 ppm after 24 hours, and the optimal growth of bacteria was observed at zinc concentrations of 10 and 20 ppm. In 48 and 72 hours the MIC was 50 ppm, while the optimal growth rate of bacteria was observed at 10 and 20 ppm. In addition, the bacteria examined in three different times had a similar growth pattern, and the highest degree of sensitivity of the strains appeared in the first 24 hours (Figure 3). The detection of *nifH* gene in DNA extracted from all *A. chroococcum* strains is indicative of the presence of the nitrogenase system in these bacteria (Figure 4).

**Table 1.** *A. chroococcum* Identification Tests ^a^

Bacterial Strains	Cat	Ox	Man F	Cys F	N_2_ Mic	St F	Nit R	Glu F	Sac F	In F	BC Fo	Mov	Capy F	Capr F	Rh F
**NFM1**	+	+	+	+	-	-	+	+	+	-	+	+	-	+	-
**NFM2**	+	+	+	+	-	-	+	+	+	-	+	+	-	+	-
**NFM3**	+	+	+	+	-	-	+	+	+	-	+	+	-	+	-
**NFM4**	+	+	+	+	-	-	+	+	+	-	+	+	-	+	-
**NFM5**	+	+	+	+	-	-	+	+	+	-	+	+	-	+	-
**NFM6**	+	+	+	+	-	-	+	+	+	-	+	+	-	+	-
**NFM7**	+	+	+	+	-	-	+	+	+	-	+	+	-	+	-
**NFM8**	+	+	+	+	-	-	+	+	+	-	+	+	-	+	-
**NFM9**	+	+	+	+	-	-	+	+	+	-	+	+	-	+	-
**NFM10**	+	+	+	+	-	-	+	+	+	-	+	+	-	+	-
**NFM11**	+	+	+	+	-	-	+	+	+	-	+	+	-	+	-
**NFM12**	+	+	+	+	-	-	+	+	+	-	+	+	-	+	-

^a^ Abbreviations: B C Fo, brown colony formation; Mov, movement; Cap F, caproate fermentation; Capy F, caprylate fermentation; Cat, catalase; Cys F, cyst formation; Glu F, glucose fermentation; In F, innositol fermentation; Man F, manitol fermentation; N_2_ Mic, nitrogen fixation in a microaerophil condition; Ox, oxidase; Rh F, rhamnose fermentation; Sac F, saccharose fermentation; St F, starch fermentation; Nit R, nitrate reduction.

## 5. Discussion

In this study, in addition to the isolation and identification of nitrogen-fixing soil-borne bacteria, the zinc-sensitivity of the different strains as well as the presence of *nifH* gene in the isolated strains were investigated. Of a total of 12 isolated *A. chroococcum* strains, all samples were capable to grow on a zinc-free medium as well as media containing a zinc concentration of 6-40 ppm, but not media with a zinc concentration of 50 ppm. These results are in line with those of Chengqun and Huang who used a nitrogen-free medium supplied with manitol, as a carbon source, to isolate Azotobacteria from pine rhizosphere (13). Babich and Stotzky studied the effects of zinc on soil-borne bacteria and reported that 2 mmol of zinc reduces the activity of bacteria (14). Cevik and Karaca reported that bacteria in pot soil are sensitive to 50 mg/kg zinc (15). In the present study, we determined a threshold value of 20 ppm for soil Zn to allow different strains of *A. chroococcum* to survive, while concentrations more than 20 ppm resulted in a linear reduction of growth, with a complete inhibition of growth at 50 ppm.

In a study by Shakibaei et al., bacteria were sensitive to Zn at 30 ppm (16), a finding that is in line with our results. Malakootian and Toolabi studied the sensitivity of waste water-borne bacteria to ZnO nanoparticles and showed that the bacteria were not sensitive to an 80 ppm concentration, while 100 and 1000 ppm concentrations resulted in 36% and 84% bacterial death, respectively (17). Our results are not in line with those of the latter study, and the difference could be due to the difference in the sampling locations as well as type of the microorganisms sampled and type of Zn metal used. Rajapaksha et al. have also shown that an increasing concentration of Zn for a short period of time linearly reduces the population size of soil-borne bacteria (18). 

Our PCR results revealed the presence of *nifH* gene in all 12 *A. chroococcum* strains (6, 19, 20). The *nifH* gene product serves as a component of the nitrogenase system and has a role in the formation of Fe-protein complex. It is therefore, safe to assume that the presence of *nifH* gene is an indicator of the existence of the nitrogenase system and the ability to fix molecular nitrogen (10). 

In conclusion, the results of the present study demonstrated that of the 12 isolated *A. chroococcum* strains, all were sensitive to a 50 ppm concentration of zinc, and that the optimum concentration of zinc for the growth of these bacteria is 20 ppm. According to our results, it appears that the optimal activity and growth of the isolated *A. chroococcum* strains are in the presence of a 6-20 ppm zinc concentration. All these strains carried *nifH* gene, which is an indicator of the nitrogenase enzyme system, and nitrogen fixing capability.
